# Chronic Rhinosinusitis with Nasal Polyps: Age and Disease Severity Differences in the Levels of Inflammatory Markers

**DOI:** 10.3390/medicina57030282

**Published:** 2021-03-18

**Authors:** Justinas Vaitkus, Astra Vitkauskienė, Regimantas Simuntis, Žygimantas Vaitkus, Nora Šiupšinskienė, Saulius Vaitkus

**Affiliations:** 1 Department of Otorhinolaryngology, Medicine Academy, Lithuanian University of Health Sciences, LT-50161 Kaunas, Lithuania; norai_s@yahoo.com (N.Š.); saulius.vaitkus@gmail.com (S.V.); 2 Department of Laboratory Medicine, Medicine Academy, Lithuanian University of Health Sciences, LT-50161 Kaunas, Lithuania; astra.vitkauskiene@kaunoklinikos.lt; 3 Department of Oral and Maxillofacial Surgery, Medicine Academy, Lithuanian University of Health Sciences, LT-50161 Kaunas, Lithuania; rsimuntis@yahoo.com; 4 Medicine Academy, Lithuanian University of Health Sciences, LT-50161 Kaunas, Lithuania; zygimantas.vaitkus@gmail.com; 5 Faculty of Health Sciences, Klaipeda University, LT-92294 Klaipeda, Lithuania

**Keywords:** chronic rhinosinusitis, nasal polyps, severity of disease, age, inflammatory markers

## Abstract

*Background and objectives:* The aim of our study was to analyze the concentrations of inflammatory markers in the nasal tissue of patients with chronic rhinosinusitis with nasal polyps (CRSwNPs) and controls of different age groups, as well as to find associations between age, inflammation development, and NPs. *Materials and methods*: Patients were divided into two groups—patients with CRSwNPs and control subjects who had nasal surgery for another reason beside CRS. Our analysis was performed across three different age groups (18–30 years, 31–50 years, and 51 years and more). Tissue biopsies from the sinus cavity for all study participants were taken and frozen at −80 °C, until use. The concentrations of IL-1β, IL-2, IL-4, IL-5, IL-6, IL-7, IL-10, IL-13, IL-21, and IL-22, were quantified using a magnetic bead-based multiplex assay. *Results*: In the group aged 18–30 years, the levels of inflammatory markers IL-1, IL-2, IL-5, and IL-22 were significantly higher in patients with CRSwNPs than the control subjects. Among patients aged 31–50 years, significantly higher concentrations of IL-2, IL-4, IL-5, and IL-22 were recorded in patients with CRSwNPs, as compared to the control subjects. In the oldest group (aged 51 years and more), patients with CRSwNPs had significantly higher concentrations of IL-2, IL-4, and IL-22, as compared to the control group. In the CRSwNP group, only the concentration of IL-21 was significantly higher among patients aged 31–50 years, as compared with those aged 51 years and older (*p* = 0.013). *Conclusions:* IL-2 and IL-22 levels were significantly higher in patients with CRSwNP than the control, across all age groups. Only the concentration of IL-21 was higher among patients with CRSwNP in the middle age group, as compared to the oldest group. IL-2, IL-4, and IL-22 levels correlated with the severity of CRSwNPs. Elevated concentrations of IL-2, IL-4, and IL-22 were determined in patients’ groups with higher sinonasal outcome test (SNOT-22) scores, pointing to more severe clinical symptoms.

## 1. Introduction

Chronic rhinosinusitis (CRS) is an inflammation of the paranasal sinuses that typically lasts beyond 12 weeks. The exact cause of benign nasal polyps (NPs) is unknown. Multiple factors are associated with CRS development, including microorganisms, allergic, and nonallergic immunologic inflammation, as well as noninfectious and nonimmunologic causes such as abnormalities in leukotriene production or responsiveness, nociceptive dysfunction, or local irritation caused by gastroesophageal reflux, defects in mucociliary clearance, and aspirin-associated respiratory disease [1]. NPs are believed to arise in the nasal mucosa due to chronic inflammation. Males are affected more often than females; adults are affected more often than children. The nasal microbiota of co-subjects is different from that of CRS with NPs (CRSwNPs) without asthma and CRSwNPs with co-morbid asthma patients [2].

Environmental factors such as inhaled pollutants such as cigarette smoke are found to play a significant role in the development of diseases of the upper airway, including CRS. Active tobacco smoking is associated with an increase in systemic inflammation markers among patients with CRS [3]. Nasal mucosal immune reactivity might occur at varying degrees in polyps with allergic and nonallergic rhinitis, but exposure to cigarette smoke is a major risk factor for airway inflammation. Multiple studies analyzed the pathophysiological effects of tobacco smoke on sinonasal mucosa. Cigarette smoke induces a physiological nasal response, including increased nasal airway resistance, nasal irritation, nasal congestion, and rhinorrhea [4,5,6].

Several studies show that the inflammatory marker concentration differs compared to the CRSwNP and control groups [7,8].

In a clinical setting, it is important to measure interleukin expression differences in order to determine possible endotypes for CRSwNP patients. This is because chronic rhinosinusitis might be driven by various pathomechanisms such as type 1 (INF-γ), type 2 (IL-4, IL-5), and type 3 (IL-17 family). In addition, it is very important to differentiate clinical phenotypes with endotypes because different phenotypes might be characterized by one endotype. Understanding this differentiation might help to expand treatment options and to suggest effective personalized treatment [9].

The prevalence of CRS is associated with patients’ age. Chronic sinusitis with polyposis is rarely diagnosed at a young age. The prevalence of CRS sharply increases after the age of 50 years. Patients aged 60 years and older are twice as likely to have CRS than those aged 19–39 years. There is an insufficient amount of data regarding the potential appearance of inflammatory changes in CRS polyp tissue across different patient age groups.

A large cohort study in the United States reported that the incidence of CRSwNPs in the age range of 65–74 years was almost two-fold higher, as compared to the CRS without NPs (CRSsNPs) or control subjects [10]. CRSwNP in the elderly is not well-investigated, despite the disease burden to geriatric patients and its impact on other common geriatric problems [11]. In Denmark, the estimated incidence of nasal polyposis increases with age, reaching a peak in the age group of 40 to 69 years and with a very small incidence in children and oldest patient groups (>80 years old) [12]. There are differences in the histopathology of CRS between adults and children; the tissue of pediatric patients with CRS tends to have higher levels of lymphocytes, monocyte/macrophages, neutrophils, and natural killer cells, as well as fewer submucosal glands, thinner epithelium, and lower eosinophils infiltration in mucosa and submucosa, when compared with the tissue of adults with CRS [13]. This might suggest that if inflammation differs between children and adults, it might also differ across different age groups in the adult population.

The aim of our study was to analyze the concentrations of inflammatory markers in the nasal tissue of patients with CRSwNPs and different age control groups.

## 2. Materials and Methods

### 2.1. Patients’ Characteristics

A total of 111 patients who were treated in the Department of Otorhinolaryngology, Hospital of the Lithuanian University of Health Sciences Kauno Klinikos between 2017–2019 years, were enrolled in this study. The study group comprised 59 patients with CRSwNPs who underwent endonasal sinus surgery and 52 controls who underwent other rhinological surgeries (those involving the skull base or lacrimal duct, orbital decompression surgery, or septoplasty), but had no history of any sinonasal diseases or recent trauma. In the CRSwNP group, mucosa was taken from the polyp tissue and in the control group, mucosa was taken from middle turbinate. Chronic rhinosinusitis was diagnosed according to the international EPOS 2012 consensus [14]. The present study was conducted as part of a larger study whose primary aim was to investigate cytokine level changes comparing CRSwNP patients and control groups with other disease-specific factors [7].

The Lund-MacKay scores were used while evaluating sinus computed tomography scans (CT). When reading a CT scan of the paranasal sinuses and ostiomeatal complex, a reader assigned each sinus the following score—0 (no abnormality), 1 (partial opacification), or 2 (complete opacification). The ostiomeatal complex was assigned a score of either 0 (not obstructed) or 2 (obstructed). Each side was graded separately. A combined score of up to 24 was possible [15].

The severity of symptoms was evaluated with the Lithuanian version of the sinonasal outcome test (SNOT-22). It is a simple disease-specific questionnaire consisting of 22 items reflecting the health burden of rhinology patients. Each item quantified the severity of symptoms from a score of 0 (no problem) to 5 (worst symptom). The sum of scores on all items resulted in a maximum score of 110. Higher score indicated a poorer outcome. The SNOT-22 is widely adopted in clinical practice and was declared as the most suitable scoring system of sinonasal outcome [16].

The exclusions criteria were as follows. Age ≤ 18 years, pregnancy, any autoimmune disease, diabetes, inverted papilloma, cystic fibrosis, or sinonasal granulomatous disease. Data on demographic characteristics, medical comorbidities, and smoking history were collected using a standard questionnaire. Participants were divided into 3 age groups—18–30 years, 31–50 years, and 51 years and more. Non-smokers were defined as never smokers; otherwise, the subjects were classified as smokers (past and current smokers were included in the same group).

The study was approved by the Kaunas Regional Biomedical Research Ethics Committee (No. P1-86-2004 (originally issued in 2004 and updated in 2016), and written consent from all subjects was received. Tissue biopsies from the sinus cavity for all study participants were taken and frozen at −80 °C, until use.

### 2.2. Determination of Inflammatory Markers Concentration

Before testing, tissue (50 mg) was transferred to gentleMACS C tubes containing tissue extraction reagent Buffer AL and was homogenized using a gentleMACS homogenizer for 1–2 min, until samples were in a consistent solution. Homogenized samples were transferred to 1.5-mL tubes and centrifuged for 5 min at 10,000× *g*. After centrifugation, supernatants were diluted twice, and the concentrations of 10 different serum cytokines—interleukin-1beta (IL-1β), IL-2, IL-4, IL-5, IL-6, IL-7, IL-10, IL-13, IL-21, and IL-22—were quantified using a magnetic bead-based multiplex assay (Human Cytokine Premixed Multi-Analyte Kit, R&D, Europe) via a Luminex^®^ 100 Analyzer (Luminex Corporation, United States), according to the manufacturer’s instructions.

### 2.3. Statistical Analysis

Statistical data analysis was performed using the statistical package IBM SPSS 23.0. The Kolmogorov–Smirnov test was employed to determine the distribution of quantitative data. Data found to be non-normally distributed were expressed as the median (interquartile range, IQR) and were compared using the Kruskal–Wallis, multiple-comparison Dunn’s, and Mann–Whitney U tests.

Data found to be normally distributed were expressed as mean and standard deviation, and were compared using Student’s *t*-test.

The chi-square test and the Fisher exact test (for small samples) were used to determine whether relationships existed between qualitative data. Spearman correlation analysis was performed to determine relationships between SNOT-22 questionnaire scores and interleukin levels. Differences comparing the groups were considered statistically significant when a *p*-value was less than 0.05.

## 3. Results

The CRSwNP and control groups were matched for age, sex, and smoking status (Table 1).

**Figure 1:**
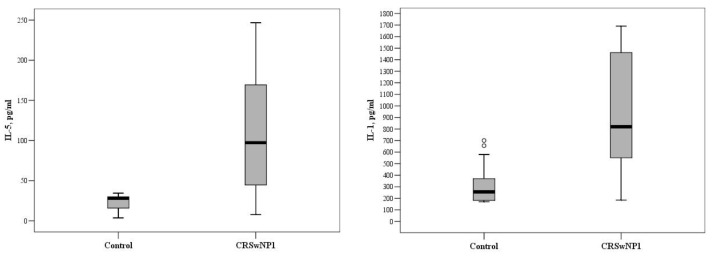
The circulating concentrations of IL-1 and IL-5 in the control and CRSwNP groups, aged 18–30 years (*p* = 0.04 and *p* = 0.032, respectively). °—indicate outliers.

In the group aged 18–30 years, the levels of inflammatory markers IL-1, IL-2, IL-5, and IL-22 were significantly higher in patients with CRSwNPs than control subjects, but the concentrations of IL-4, IL-6, IL-7, IL-10, IL-12, and IL-13 did not differ significantly between groups. The median concentrations of IL-1 and IL-5 were 820.0 (367.7–1576.0) and 97.2 (26.1–208.0) pg/mL vs. (175.3–373.5) and 28.2 (11.7–30.0) pg/mL in the CRSwNP and control groups, respectively (*p* = 0.04 and *p* = 0.032, Figure 1).

**Figure 2:**
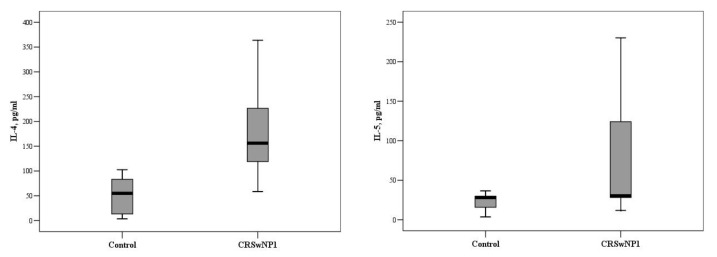
The circulating concentrations of IL-4 and IL-5 in the control and CRSwNP groups, aged 31–50 years (*p* < 0.001 and *p* = 0.014, respectively).

In the group aged 31–50 years, significantly higher concentrations of IL-2, IL-4, IL-5, and IL-22 were recorded in patients with CRSwNPs, as compared to the control group, but there was no significant difference in the concentrations of other ILs between these groups. The median concentrations of IL-4 and IL-5 were 54.8 (10.2–84.1) and 28.2 (13.8–30.0) pg/mL vs. 156.4 (110.5–231.2) and 30.1 (28.2–136.4) pg/mL in the control and CRSwNP groups, respectively (*p* < 0.001 and *p* = 0.014, Figure 2).

**Figure 3:**
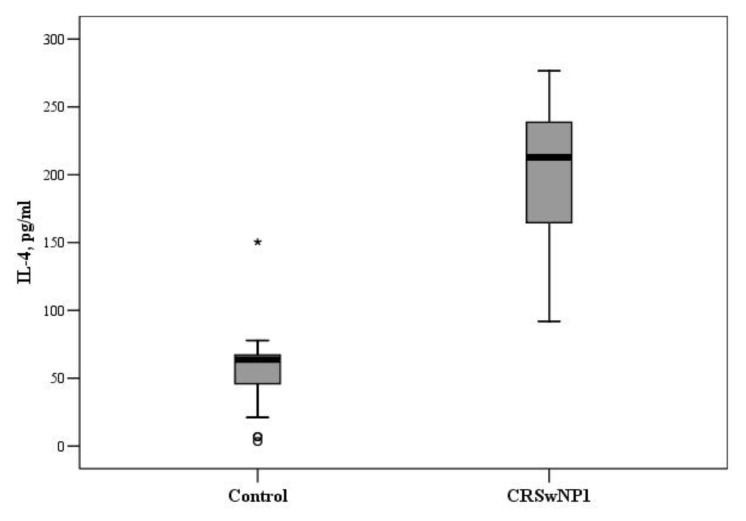
The circulating concentrations of IL-4 in the control and CRSwNP groups aged 51 years and more (*p* < 0.001). °—indicate outliers, *—indicate extreme outliers.

In the group aged 51 years and more, the concentrations of IL-2, IL-4, and IL-22 were found to be significantly higher in patients with CRSwNPs, compared with the control group. The median concentration of IL-4 was 63.7 (37.0–67.2) and 212.7 (163.7–242.0) pg/mL in the control and CRSwNP groups, respectively (*p* < 0.001, Figure 3).

**Figure 4:**
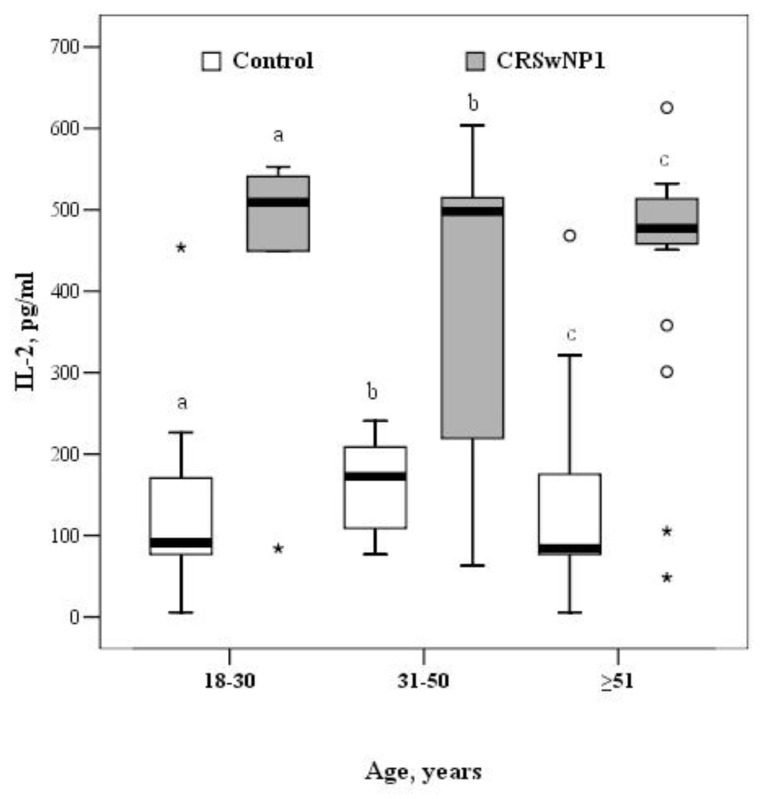
The circulating concentrations of IL-2 in the control and CRSwNP groups by different age groups (a *p* = 0.016, b *p* = 0.001, c *p* < 0.001). °—indicate outliers, *—indicate extreme outliers.

The concentrations of IL-2 and IL-22 were significantly higher in the CRSwNP than the control group, across all age groups. In the group aged 18–30 years, the median concentration of IL-2 in the control and CRSwNP groups was 91.2 (77.0–177.2) and 509.1 (266.8–546.9) pg/mL, respectively (*p* = 0.015). In the 31–50-year-old group, the median concentration of IL-2 was 172.5 (103.6–217.5) and 498.2 (213.1–518.2) pg/mL in the control and CRSwNP groups, respectively (*p* = 0.001). In the oldest group, the median concentration of IL-2 was almost 6-fold higher in the CRSwNP than the control group (477.1 (457.5–514.2) vs. 84.1 (77.0–181.3) pg/mL, *p* < 0.001) (Figure 4).

**Figure 5:**
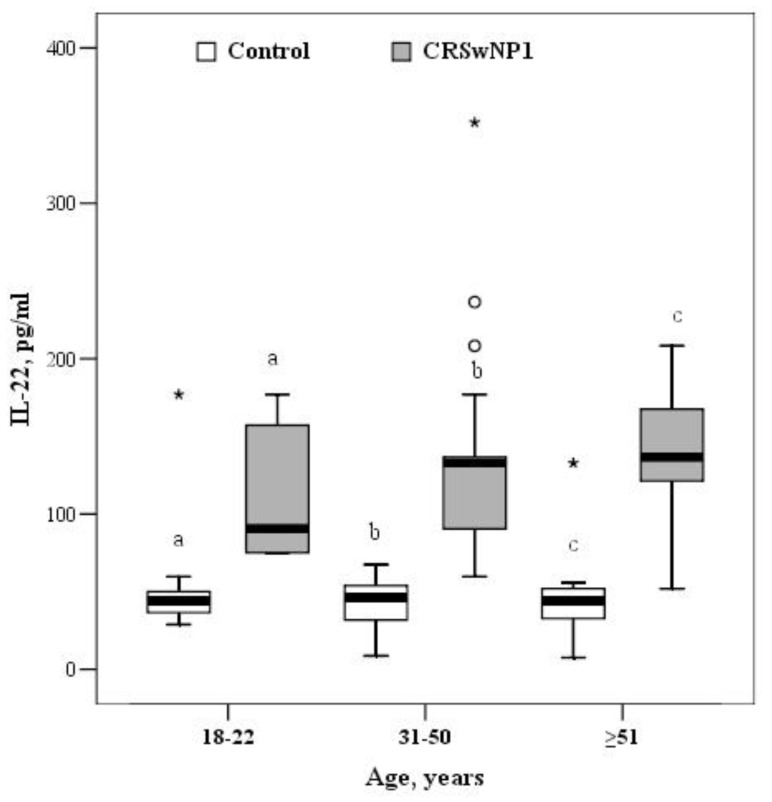
The circulating concentrations of IL-22 in in the control and CRSwNP groups by different age groups (a *p* = 0.002, bc *p* < 0.001). °—indicate outliers, *—indicate extreme outliers.

In the 18–30-year age group, the median concentration of IL-22 was 44.3 (36.6–52.0) and 90.5 (75.1–167.1) pg/mL in the control and CRSwNP groups, respectively (*p* = 0.002). Among 31–50-year olds, control patients had a significantly lower median concentration of IL-22 than patients with CRSwNP (46.2 (31.3–54.9) vs. 132.8 (90.5–146.8) pg/mL, *p* < 0.001). In the oldest group, the median concentration of IL-22 was 3-fold greater in patients with CRSwNP than the controls (136.7 (115.5–167.5) vs. 44.3 (32.7–52.0) pg/mL, *p* < 0.001) (Figure 5).

Comparison of IL concentrations in patients with CRSwNP across different age groups revealed no significant differences in the concentrations of all ILs except for the concentration of IL-21 being significantly higher in 31–50-year old patients than those aged 51 years and older (Table 2).

**Figure 6:**
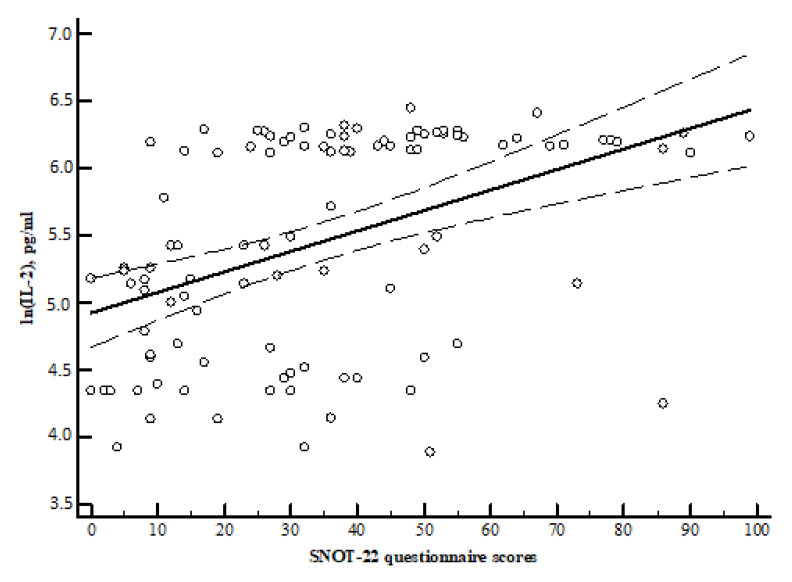
r = 0.464, y = 4.9231 + 0.01522x. r—Spearman correlation coefficient. x = SNOT-22 questionnaire scores sum. y = interleukin IL-2 pg/mL.

**Figure 7:**
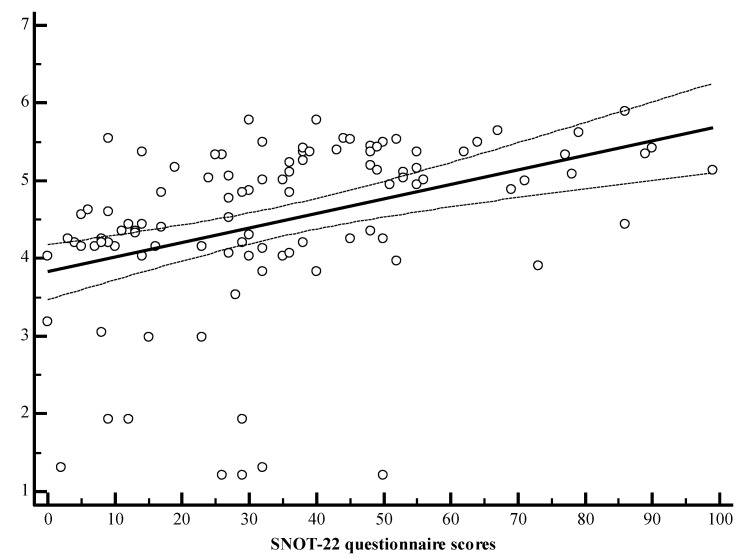
r = 0.502, y = 3.8265 + 0.01876x. r—Spearman correlation coefficient. x = SNOT-22 questionnaire scores sum. y = interleukin IL-4 pg/mL.

**Figure 8:**
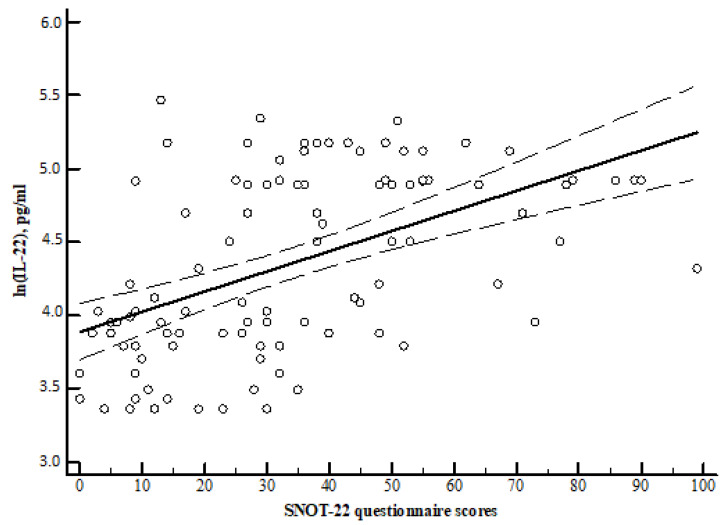
r = 0.521 y = 3.8904 + 0.01373x. r—Spearman correlation coefficient. x = SNOT-22 questionnaire scores sum. y = interleukin IL-22 pg/mL.

Spearman correlation analysis showed that the SNOT-22 questionnaire scores were directly and significantly correlated with the concentrations of IL-2, IL-4, and IL-22 (Table 3, Figure 6, Figure 7 and Figure 8).

For comparison, the Mann–Whitney U test was applied. The horizontal lines represent the medians; the circles represent the outliers.

For comparison, the Mann–Whitney U test was applied. The horizontal lines represent medians.

For comparison, the Mann–Whitney U test was applied. The horizontal lines represent medians. The circles represent outliers; the asterisks represent extreme outliers.

For comparison, the Mann–Whitney U test was applied. The horizontal lines represent medians. The circles represent outliers; the asterisks represents extreme outliers.

For comparison, the Mann–Whitney U test was applied. The horizontal lines represent medians. The circles represent outliers; the asterisks represent extreme outliers.

## 4. Discussion

Pettiford’s found that distinct age-related differences in the human response to acute illness might be useful in directing future efforts at immunomodulatory therapies [17]. It is known that nasal polyps rarely develop at a young age and are more commonly found in elderly patients, and so we hypothesized that this might be influenced by changes in immune response, which affects the course of CRS.

In our study, the mean age of patients was 50.3 ± 14.7 years. The frequency of asthma was significantly higher in CRSwNP than in the control group. The study by Won et al. showed CRSwNP to be significantly associated with adult-onset asthma (onset after 18 years of age) or late-onset asthma (onset after 40 years of age), whereas CRSsNPs was related to childhood-onset asthma (onset before 18 years) or early-onset asthma (onset before 40 years) in adults [18]. This study also reported distinct age-related patterns of CRSwNPs and asthma and demonstrated their significant associations in the general population [18]. In addition, Loftus et al. showed that CRSwNP patients with comorbid asthma (22%) statistically significantly underwent revision surgery more often than those without comorbid asthma (8%) [19]. Therefore comorbid asthma might be one of the criteria to admit CRSwNP patients for monoclonal antibodies treatment, as the EPOS2020 steering group concluded [20].

Ahern and Cervin confirmed that the inflammatory state of CRS is highly heterogeneous, with mixed profiles of type 1, 2, and 3 inflammation seen within classical CRSsNP and CRSwNP phenotypes. Endotyping of a CRS disease state emerged as a useful tool in identifying key inflammatory profiles amongst patients with CRS, and provides a unique opportunity for targeted treatment options [21]. Our study showed significant differences in the levels of inflammatory markers between control subjects and patients with CRSwNPs across all age groups. To our knowledge this is the first study in Europe to find associations between IL levels and age, in adults with CRSwNPs. In the youngest group, i.e., 18–30 years old, we found mixed profiles of type 1, 2, and 3 cytokines. The majority of changes in IL concentrations were observed in the middle-aged group of patients with CRSwNPs compared to the control subjects. The concentrations of IL-2, IL-4, IL-5, and IL-22 were significantly higher in the tissues of NPs than control tissues in the 31–50-year old group. Patients with CRSwNP in the group of 50 years and older had significantly higher concentrations of only three different ILs (IL-2, IL-4, and IL-22). Different interleukins were identified in different age groups that could be prognostically important in the assessment of chronic sinusitis. According to prevailing inflammation, it might allow one to pick the most effective individual treatment. Morse et al. reported that patients aged 60 years and more had increased mucus levels of IL-1b, IL-6, IL-8, and TNF-a, when compared to their younger counterparts [22].

Increased levels of proinflammatory cytokines were associated with both tissue neutrophilia and symptomatic bacterial infection/colonization in aged patients. They identified 5 different endotypes. One endotype that was more specific for patients aged 60 years and older was associated with elevated IL-1b, IL-6, IL-8, and TNF-a, i.e., cytokines linked with activation of the body’s innate immune system in the setting of both acute and chronic inflammation [23]. A similar inflammatory pattern was identified in the overall cohort, with relatively consistent levels of these pro-inflammatory cytokines in younger patients that subsequently increased longitudinally after the age of 60 years. Aged patients with CRS have a unique inflammatory signature that corresponds to a neutrophilic proinflammatory response. Neutrophil-driven inflammation in aged patients with CRS might be less likely to respond to corticosteroids and might be closely linked to chronic microbial infection or colonization [23].

The results of the study by Hwang et al. suggested that the elderly population has distinct pathophysiology. Clinical presentation from adults with CRS and management of elderly patients (>65 years old) with CRS might require different or additional therapeutic approaches [24]. However, our study showed that among patients with CRSwNPs, only the concentration of IL-21 was higher in the middle age group than in the group aged 51 years and more. Concentrations of other ILs were significantly higher when the control and CRS groups were compared, but we noticed no difference when comparing IL concentrations between different CRS age groups.

## 5. Conclusions

IL-2 and IL-22 levels were significantly higher in CRSwNP patients’ tissues compared with tissues obtained from adults across all age groups in the control groups. Only the concentration of IL-21 was higher among patients with CRSwNP in the middle age group as compared to the oldest group. IL-2, IL-4, and IL-22 levels correlated with the severity of CRSwNPs. Elevated concentrations of IL-2, IL-4, and IL-22 were determined in patients groups with higher SNOT-22 scores pointing to more severe clinical symptoms.

## Figures and Tables

**Table 1:** Characteristics of the patients included in the study. CRSwNP—chronic rhinosinusitis with nasal polyps.

Characteristic	CRSwNP *N* = 59 (53.2%)	Control *N* = 52 (46.8%)	*p* Value
Age, mean ± SD, years	50.3 ± 14.7	45.8 ± 19.5	0.177 *
Age groups, years			
18–30	5 (8.5)	15 (28.8)	0.02
31–50	22 (37.3)	16 (30.8)	0.235
>51	32 (54.2)	21 (40.4)	0.072
Sex			
Male	31 (52.5)	28 (53.8)	0.891
Female	28 (47.5)	24 (46.2)	
Smoking status			
Yes	11 (18.6)	4 (7.7)	0.092
Allergy			
Yes	15 (25.4)	0	<0.001
Asthma			
Yes	18 (30.5)	0	<0.001

Values are number (percentage) unless indicated otherwise. *p* value of the chi-square test; * *p* value of Student’s *t*-test.

**Table 2:** The circulating concentrations of interleukins in patients with CRSwNP for different age groups. Il—interleukin.

Marker, pg/mL	Age Groups, Years			df = 2 χ2; *p* Value
	18–30	31–50	>51	
IL-1	820.0 (367.7–1576.0)	1181.6 (864–1489.2)	1066.0 (719.3–1398.3)	0.958; 0.619
IL-2	509.1 (266.8–546.9)	498.2 (213.1–518.2)	477.1 (457.5–514.2)	0.314; 0.855
IL-4	169.7 (64.5–195.7)	156.4 (110.5–231.2)	212.7 (163.7–242.0)	4.464; 0.107
IL-5	97.2 (26.1–208.0)	30.1 (28.2–136.4)	29.9 (24.1–32.3)	3.495; 0.174
IL-6	208.3 (147.1–242.0)	180.9 (150.3–233.5)	209.0 (157.5–233.5)	0.361; 0.835
IL-7	22.8 (16.4–122.8)	33.7 (29.4–123.4)	29.9 (23.1–47.2)	4.305; 0.116
IL-10	190.1 (119.0–198.4)	258.8 (171.4–396.7)	233.9 (119.0–348.0)	4.508; 0.105
IL-13	165.2 (154.5–199.3)	145.3 (121.4–168.2)	160.2 (135.9–169.2)	3.207; 0.201
IL-21	109.6 (74.6–144.2)	112.2 * (97.7–118.5)	92.1* (71.0–105.6)	6.29; 0.043; * *p* = 0.013
IL-22	90.5 (75.1–167.1)	132.8 (90.5–146.8)	136.7 (115.5–167.5)	1.73; 0.421

Data are median (interquartile range). Data were compared using the Kruskal–Wallis test (multiple comparison by Mann–Whitney test). Significant multiple comparison marked by *.

**Table 3:** Correlation between sinonasal outcome test (SNOT-22) questionnaire scores and interleukin (IL) levels.

**The sum of the SNOT-22 Questionnaire Scores**	**Interleukins**		
	IL-2	IL-4	IL-22
	r = 0.464, *p* < 0.001	r = 0.502, *p* < 0.001	r = 0.521, *p* < 0.001

r—Spearman correlation coefficient.

## Data Availability

All data relevant to the study are included in the article.
